# La educación virtual en odontología

**DOI:** 10.21142/2523-2754-0904-2021-079

**Published:** 2021-12-09

**Authors:** Denisse Pilar Carmen Aguilar Gálvez

**Affiliations:** 1 División de Odontopediatría, Carrera de Odontología de la Universidad Científica del Sur. Lima, Perú. daguilar@cientifica.edu.pe Universidad Científica del Sur División de Odontopediatría Carrera de Odontología Universidad Científica del Sur Lima Peru daguilar@cientifica.edu.pe

La enseñanza de la odontología tradicional, en todas sus especialidades, se ha basado siempre en el traspaso del conocimiento de manera presencial, es decir, un tutor enseña cómo realizar el procedimiento y el alumno repite lo indicado. Del mismo modo, a lo largo de los años se ha llevado a cabo la enseñanza del diagnóstico basado en signos y síntomas identificados de manera presencial, es decir, con el paciente frente a los clínicos ^1^. Sin embargo, desde el año 1998, se ha presentado una tendencia a la simulación de este proceso de aprendizaje ^2^. Esta simulación incluye el uso de simuladores físicos que permiten sentir, tocar y realizar procedimientos odontológicos como colocar una anestesia dental. Asimismo, los simuladores pueden emitir sonidos de incomodidad o malestar si el procedimiento no está bien hecho, o incluso se puede observar un diente sangrando cuando la preparación cavitaria alcanza la pulpa dental, entre otros procedimientos. En la última década, este enfoque de enseñanza ha evolucionado con la aparición de *software* de simulación que incluye textos, gráficos y animaciones, por lo que, en muchos casos, no resulta necesario el espacio físico para unidades o fantomas, y si se dispone de un laboratorio con computadoras como el sistema Mood, una sola unidad puede contener información de simulación de más de 100 tratamientos que permiten un aprendizaje idóneo para los estudiantes ^3^.

En el contexto actual, que incluye la pandemia mundial por COVID-19 y el planteamiento de un esquema de enseñanza bimodal, es preciso implementar un ambiente virtual de aprendizaje que cuente con una plataforma y esté vinculado con objetos virtuales de aprendizaje y todo lo que implica este tipo de enseñanza. Esta implementación asegurará una forma de aprendizaje eficiente y completa que beneficiará a los estudiantes ^4^. Sin embargo, la literatura científica odontológica sobre este esquema de aprendizaje es escasa, por lo que se necesitan más estudios que, seguramente, confirmarán la utilidad de estos sistemas de aprendizaje ^5^.
